# 3D-Printed Poly(lactic acid)/Poly(ethylene glycol) Scaffolds with Shape-Memory Effect near Physiological Temperature

**DOI:** 10.3390/polym18010140

**Published:** 2026-01-03

**Authors:** Anastasia A. Fetisova, Abdullah bin Firoz, Alexandr S. Lozhkomoev, Elena I. Senkina, Egor E. Ryumin, Maria A. Surmeneva, Roman A. Surmenev

**Affiliations:** 1 International Research and Development Center Piezo- and Magnetoelectric Materials, Research School of Chemistry and Applied Biomedical Sciences, National Research Tomsk Polytechnic University, 30 Lenina Avenue, Tomsk 634050, Russia; aaf71@tpu.ru (A.A.F.); surmenevamaria@tpu.ru (M.A.S.); 2 Physical Materials Science and Composite Materials Centre, Research School of Chemistry and Applied Biomedical Sciences, National Research Tomsk Polytechnic University, 30 Lenina Avenue, Tomsk 634050, Russia; er.abdullahbinfiroz@gmail.com (A.b.F.); asl@ispms.ru (A.S.L.); 3 Institute of Strength Physics and Materials Science of Siberian Branch of the Russian Academy of Sciences (ISPMS), 2/4 Akademicheskii Pr., Tomsk 634055, Russia; elena.senkina.1995@mail.ru (E.I.S.); ryuminee@ispms.ru (E.E.R.)

**Keywords:** shape-memory polymer, 3D printing, additive manufacturing, tissue engineering

## Abstract

Biocompatible poly(lactic acid) (PLA) was plasticized with poly(ethylene glycol) (PEG) added at concentrations of 10, 15, and 20 wt.% relative to PLA, and then processed into gyroid triply periodic minimal surface (TPMS) scaffolds using fused filament fabrication (FFF) 3D printing. The influence of PEG concentration and gyroid structure (50% infill density) on thermal transitions, crystallinity, and low–temperature shape-memory performance was systematically investigated. The shape-memory effect (SME) of the PLA–based scaffolds was tailored through compositional control and structural design. Shape recovery under thermal activation at 40 °C and 50 °C was examined to reveal the correlation between composition and structure in governing low–temperature shape-memory behavior. The optimal composition (PLA/10 PEG, 50% gyroid infill) achieved shape recovery with a recovery ratio (*R*_r_) of 97 ± 1% at 40 °C within 6 ± 1 min, demonstrating optimal shape-memory activation close to physiological temperature. Structural and morphological changes were characterized using ATR–FTIR, Raman spectroscopy, DSC, XRD, and SEM, providing comprehensive insight into the plasticization of the PLA matrix and its impact on structure–property relationships relevant to bone tissue engineering.

## 1. Introduction

Shape-memory polymers (SMPs) are attractive for biomedical devices and 3D-printed structure because they can be temporarily deformed and subsequently recover a permanent shape when exposed to an external stimulus, typically heat. Lendlein and Langer first demonstrated that biodegradable thermoplastic SMPs could combine elastic recovery with controlled degradation, opening the way for minimally invasive implants and self–deploying scaffolds [1]. Among available SMPs, PLA is particularly appealing for bone tissue engineering because it is bio–based, bioresorbable, and readily processed by material extrusion 3D printing. However, its relatively high glass transition temperature (*T*_g_ ≈ 55–65 °C) and intrinsic brittleness limit shape-memory activation at physiologically relevant temperatures [2]. Comprehensive reviews of 3D-printed PLA highlight that lowering the *T*_g_ and tailoring the internal structure are essential to achieve rapid, reversible actuation near body temperature [3,4].

A widely used strategy to decrease PLA’s *T*_g_ and improve ductility is blending with PEG [5]. PEG acts as a hydrophilic plasticizer, decreasing the *T*_g_ and cold crystallization temperature (*T*_cc_), enhancing chain mobility, and increasing surface wettability [6]. Serra et al. showed for 3D-printed PLA/PEG scaffolds that 5–20 wt.% PEG not only improves processability but also produces thicker printed filaments (struts), and a smaller pore size, accelerates hydrolytic degradation, and strongly modifies crystallinity [7].

O’Mahony et al. systematically linked the thermal and thermomechanical properties of PLA blends to their suitability for FFF, demonstrating that plasticizer content governs the usable processing window for extrusion temperatures, cooling profiles, and build chamber conditions [8]. Shin et al. reported that increasing PEG concentration in melt–extruded PLA/PEG films reduces *T*_g_ and *T*_cc_, improves crystallization, and yields a transition from brittle to ductile behavior; however, excessive PEG content promotes phase separation and a loss of strength [9]. More recently, Kumar et al. studied 3D-printed PLA/PEG parts and showed that 1–5 wt.% PEG decreases tensile modulus and strength but markedly increases elongation at break, impact resistance, and surface hydrophilicity, identifying 5 wt.% as a good compromise between printability and mechanical performance [6]. Altogether, these studies demonstrate that PEG concentration has a strong effect on melt viscosity and solid–state structure, and therefore requires composition–dependent optimization of processing parameters in any reproducible 3D–printing protocol.

Similarly, multiple studies have analyzed the shape-memory response of 3D-printed PLA structures. Senatov et al. used FFF to produce porous PLA/hydroxyapatite scaffolds and demonstrated that the SME can provide “self–fitting” behavior for bone implants, with *R*_r_ of 96% after two compression–heating cycles [10]. Wu et al. demonstrated that activation temperature is the dominant parameter controlling the SME of 3D-printed PLA, while layer thickness and raster angle play secondary roles [11]. More recently, studies on 3D-printed PLA components have emphasized that operational parameters such as nozzle temperature, printing speed, and layer height must be carefully adjusted for each material formulation to ensure dimensional accuracy and consistent actuation behavior [3].

In 3D-printed scaffolds, the structure is primarily defined by the infill pattern and density, which influence both mechanical and shape-memory behavior. Ehrmann and Ehrmann systematically varied infill density and pattern in FDM-printed PLA cubes and observed that gyroid patterns offer superior shape-memory recovery but lower load-bearing capacity compared with honeycomb infills. Intermediate infill density sometimes resulted in the poorest recovery due to stress localization, and the warm water used for recovery could not readily reach all the regions of the sample [12]. Similar work on PLA–based porous scaffolds confirms that porosity and interconnectivity control mechanical stiffness, and heat transfer, both of which influence the kinetics and uniformity of shape-memory recovery [10]. Nevertheless, most published studies on PLA/PEG blends have been conducted on bulk samples or simple lattice structures, and often focus on thermal or mechanical properties rather than on systematic evaluation of shape-memory performance as a function of infill density.

For bone tissue engineering, TPMS structures such as the gyroid are particularly attractive because they combine high surface-to-volume ratios with continuous, gently curved struts that distribute stress more homogeneously and facilitate cell ingrowth and nutrient transport. Myers et al. optimized FDM parameters for PLA gyroid and Schwarz primitive lattices, showing that such structures can achieve compressive properties within the range of human trabecular bone when printed with appropriate process settings [13]. Scaffold design studies have established that bone ingrowth and vascularization are favored by porosities of roughly 50–70%, which balance mechanical stability with sufficient space for tissue ingrowth and fluid transport [14]. Within this framework, samples printed with 100% infill density serve as dense reference materials in which the effect of internal porosity is removed. In the present study, we therefore consider PLA and PLA/PEG blends printed either as fully dense samples with 100% infill and gyroid TPMS scaffolds with 50% infill density.

Another critical design requirement for biomedical SMPs is the activation temperature. For PLA–based systems, the activation temperature is usually tied to *T*_g_, so pure PLA tends to require activation well above body temperature. Plasticization and blending strategies have therefore been pursued to shift the effective transition into a clinically useful range near physiological conditions. Electrospun PLA fibers plasticized with lactic acid oligomer show excellent thermally activated SME at 45 °C and even 40 °C, demonstrating that suitably designed PLA–based systems can recover fully at temperatures only slightly above 37 °C [15]. Recent work on plasticized PLA/thermoplastic starch blends has further confirmed efficient SME at 45 °C when the *T*_g_ is depressed to a range close to physiological temperature [16]. At the same time, studies on 3D-printed PLA parts indicate that higher activation temperatures accelerate the shape recovery rate and improve the *R*_r_, albeit at the cost of deviating from strictly physiological conditions [11]. Against this background, 40 °C represents a practically relevant lower bound corresponding to mild thermal stimulation close to clinically used mild–hyperthermia regimes [17], while 50 °C can be viewed as an upper–bound condition where recovery is faster and more complete but still within the range commonly used in in vitro SMP testing [18]. Together, these two temperatures allow one to map the trade–off between clinically gentle activation and maximized SME performance in PLA/PEG scaffolds.

Despite extensive work on PLA/PEG blends and on 3D-printed PLA-based SMPs, several important gaps remain. First, the combined influence of material composition and gyroid infill density on low–temperature shape-memory behavior under aqueous activation has not been systematically addressed. Previous SMP studies have either used dense samples, simple rectilinear infills, or activation temperatures well above 50 °C; therefore, the specific contribution of open–porous gyroid structure to heat transfer and recovery kinetics near physiological temperatures remains unclear [12]. Second, quantitative comparisons of shape-memory performance between dense and porous scaffolds printed from the same PLA/PEG blends are scarce, leaving open the question of how much infill density itself enhances or compromises shape recovery ratio, recovery rate, and structural durability.

This study aims to address these gaps by (i) preparing PLA/PEG blends with 10, 15, and 20 wt.% PEG, (ii) processing them into 3D-printed scaffolds with 100% and 50% gyroid infill using FFF, and (iii) correlating PEG content and infill density with thermal transitions, crystallinity, morphology, and thermally activated shape-memory behavior at 40 °C and 50 °C. By directly comparing dense and gyroid scaffolds with identical compositions and well–defined printing parameters, we clarify the effect of infill density on shape-memory performance and demonstrate how PEG concentration governs shape-memory response.

## 2. Materials and Methods

### 2.1. Materials

PLA pellets (NatureWorks LLC, Plymouth, MN, USA) were dried at 50 °C for 24 h before use, to prevent hydrolytic and thermal deterioration related to moisture during strand extrusion. After drying, the materials were stored in a desiccator. PEG flakes (*M*_n_ = 4000 g/mol, Sigma-Aldrich, Moscow, Russia) were used as received. Chloroform (Ekos, Moscow, Russia) of high chemical purity was used as the solvent for solution casting.

### 2.2. Scaffold Fabrication

PLA was blended with PEG at mass fractions of 0%, 10%, 15%, and 20 wt.%. For each composition, PLA and PEG were dissolved in chloroform in a hermetically sealed glass bottle. The resulting solution was heated in a water bath at 40 °C under continuous magnetic stirring for 24 h, until a clear homogeneous solution was obtained. Solutions were cast into Teflon molds, and solvent was allowed to evaporate in a fume hood for 48 h at ambient temperature, ultimately forming PLA and PLA/PEG films. Films were cut into small pieces and dried in an oven at 40 °C for 24 h. Residual solvent removal was confirmed by weighing the samples until a constant mass was achieved. Films were used as feedstock for strand extrusion.

Strands were produced using a single–screw extruder equipped with a 0.8 mm nozzle at 180 °C, which is within the recommended processing window and below the onset of thermal degradation for PLA/PEG blends [19]. The nozzle temperatures were selected to ensure stable extrusion and interlayer bonding while limiting process-induced PLA degradation during melt processing. PLA properties are sensitive to nozzle temperature in FFF, and stable printing is commonly achieved at a temperature near 190 °C for neat PLA [20]. In contrast, PEG acts as a plasticizer in PLA, increasing chain mobility and reducing melt viscosity, which enables reliable deposition at lower nozzle temperature [21]. The lower temperature for PLA/PEG also reduces thermal exposure of PLA, which is beneficial because PLA is susceptible to melt-processing degradation and discoloration [22]. Strand diameter was controlled by a fixed nozzle, a constant screw speed, and air cooling, and it was visually checked to avoid bubbles and necking. After the first extrusion, a slight yellow tint was observed, which increased with the PEG content, consistent with reports that yellow–brown discoloration of PLA during melt processing is a visible sign of degradation [22,23]. The extruded strand was then chopped into 2–3 mm pellets and used as feedstock for a custom pellet–fed screw extruder mounted on an FDM gantry of a modified Ultimaker 3 3D printer [24].

Gyroid scaffolds (dimensions of 30 × 10 × 1 mm^3^) were produced by FFF using a modified Ultimaker 3 gantry system equipped with the custom pellet-fed extruder. PLA scaffolds were printed at 190 °C and PLA/PEG scaffolds at 180 °C with a 0.8 mm nozzle, 60 °C bed, 0.20 mm layer thickness, 40 mm/s printing speed, and 100% flow rate. These parameters were selected based on preliminary tests to obtain continuous deposition, good interlayer adhesion, and dimensional accuracy of the gyroid structure. The CAD model of the gyroid structure was exported in STL format and processed in the OrcaSlicer 2.0.0 software for slicing. Scaffolds were printed with infill densities of 50% and 100%. Sample codes and the weight ratios of the compounds composed of PLA and PEG are summarized in Table 1.

### 2.3. Characterization

Thermal properties of the scaffolds were studied using differential scanning calorimetry (DSC, Q2000 V24.10 Build 122, TA Instruments, New Castle, DE, USA). Samples of approximately 8 mg taken from the cross–section of scaffolds were sealed in aluminum pans and scanned from 25 °C to 250 °C at a heating rate of 10 °C/min under continuous air flow (60 mL/min). *T*_g_ was determined from the midpoint of the inflection in the heat flow curve. The *T*_cc_ and melting temperature (*T*_m_) were taken as the peak temperatures of the corresponding exothermic and endothermic transitions. The enthalpies of cold crystallization (Δ*H*_cc_) and melting (Δ*H*_m_) were obtained by integrating the respective DSC peaks using standard linear baselines. The degree of crystallinity (*X*_c_) of PLA was calculated through the following Equation (1) [25]:

(1) $$X_{c}=\left(\frac{{\Delta H}_{m}-{\Delta H}_{cc}}{{\Delta H}_{m}^{0}\times \varphi }\right)\times 100\%$$

where Δ*H*_m_ is the melting enthalpy (J/g), Δ*H*_cc_ is the cold crystallization enthalpy (J/g), Δ*H*^0^_m_ is the melting enthalpy of 100% crystalline PLA taken as 93 J/g [26], and *φ* is the weight fraction of PLA in the blend.

The crystal structure of the scaffolds was examined using an X-ray diffractometer (XRD, Shimadzu XRD 7000S, Kyoto, Japan) with CuKα radiation (λ = 1.54 Å), over a 2θ range of 5–65° with a scanning speed of 5°/min at 40 kV and 30 mA. Crystallite size was calculated using Scherrer’s Equation (2):

(2) $$D=\frac{K\times \lambda }{\left(\beta \times cos\theta \right)}$$

where *K* is the Scherrer’s constant (0.9), *λ* is the wavelength of the X-ray beam used, *β* is the full width at half maximum (FWHM) of the peak, and *θ* is the Bragg angle [27].

Surface morphology and elemental composition of the scaffolds were examined using a scanning electron microscope (SEM, Apreo 2, Thermo Fisher Scientific, Waltham, MA, USA) equipped with an energy–dispersive X-ray spectroscopy (EDS) system (Octane Elect, Gatan, Pleasanton, CA, USA), operated at an accelerating voltage of 10 kV. Samples were sputter–coated with copper using a JEE–420 vacuum evaporator (JEOL, Tokyo, Japan) before analysis.

Raman spectra were recorded using an integrated system comprising a vertical optical microscope with a 20× objective and an integrated atomic force microscopy head (NTEGRA, NT–MDT, Moscow, Russia), coupled to a Raman spectrometer (nVia Raman Microscope, Renishaw, Gloucestershire, UK) with a 532 nm He–Ne laser, and an Andor detector (1024 × 400 pixels CCD).

The chemical changes in plasticized PLA/PEG scaffolds were analyzed using an IRAffinity–1S FTIR spectrophotometer (SHIMADZU, Kyoto, Japan) in attenuated total reflectance (ATR) mode with a diamond crystal. The ATR–FTIR spectra were recorded in the range of 4000–400 cm^−1^ with a resolution of 2 cm^−1^. Background spectra were collected before each measurement and subtracted to eliminate the effects of humidity and carbon contamination.

The shape-memory performance of scaffolds was evaluated using a U–shape bending test, which comprised three steps: heating and programming, cooling and fixing, and heating and recovery [28].

For programming the temporary shape, each sample (dimensions of 30 × 10 × 1 mm^3^) was immersed in distilled water at 50 °C (*T* > *T*_g_) and kept there for 1 min. The samples were then removed, bent around a 5 mm diameter glass rod into a U-shape, and held under constant pressure for 1 min to fix the temporary shape. After the external load was removed, the samples were left for 10 min, and the fixed bending angle (*θ*_fixed_) was recorded. Shape-memory recovery was induced by immersing the bent samples in distilled water at either 40 °C or 50 °C. These temperatures were selected to evaluate recovery at both the lower end of the *T*_g_ region, where partial mobility allows gradual shape-memory recovery, and closer to the upper end, where full chain mobility enables rapid and complete recovery of the initial shape.

The evolution of the bending angle during shape-memory recovery was continuously monitored by video recording, and the instantaneous unrecovered angle (*θ*_i_) was determined. Quantitative analysis of *θ*_fixed_ and *θ*_i_ was performed using ImageJ 1.53e software. Standard shape-memory parameters, including the shape fixity ratio (*R*_f_), which characterizes the ability to retain the temporary shape, and the shape *R*_r_, which represents the ability to recover the permanent shape, were calculated in accordance with widely accepted conventions from a single programming–recovery cycle using three samples per type.

The *R*_r_ and *R*_f_ were calculated using the following Formulas (3) and (4) [29]:

(3) $$R_{f}=\left(\frac{\theta_{fixed}}{\theta_{max}}\right)\times 100\%$$

(4) $$R_{r}=\left(\frac{\theta_{max}-{\;\theta }_{i}}{\theta_{max}}\right)\times 100\%$$

where *θ*_max_ is the angle after deformation, which is taken to be equal to 180 °C; *θ*_fixed_ is the angle after cooling and load removal; and *θ*_i_ is the residual unrecovered angle after heating.

The recovery process was video–recorded for accurate angle analysis and recovery time (*T*) (Videos S1–S4). Video S1 illustrates the shape-memory recovery of PLA and PLA/PEG scaffolds at 50 °C, while Video S2 demonstrates the behavior of the same scaffolds at 40 °C. Video S3 presents the shape-memory recovery of PLA_50 and PLA/PEG_50 gyroid scaffolds at 40 °C, and Video S4 illustrates their shape-memory recovery at 50 °C.

## 3. Results and Discussion

**Figure 1:**
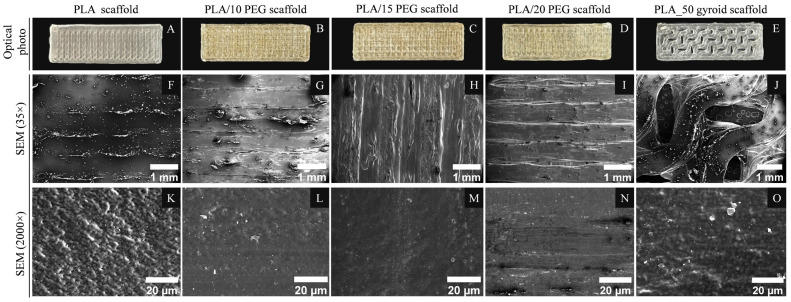
(**A**–**E**) Optical photographs and (**F**–**O**) SEM images of 3D-printed scaffolds (top–view).

SEM was used to examine the morphology of the 3D-printed scaffolds. Figure 1 shows the macroscopic and microscopic structure of PLA and PLA/PEG scaffolds. The PLA_50 and PLA/PEG_50 gyroid scaffolds displayed a pattern of elongated stripes 1 mm wide, corresponding to the path of the printing nozzle during the layer–by–layer deposition (Figure 1F–I). The gyroid structure remained well preserved after printing, with continuous, interconnected struts and open channels visible in the SEM images (Figure 1J).

**Figure 2:**
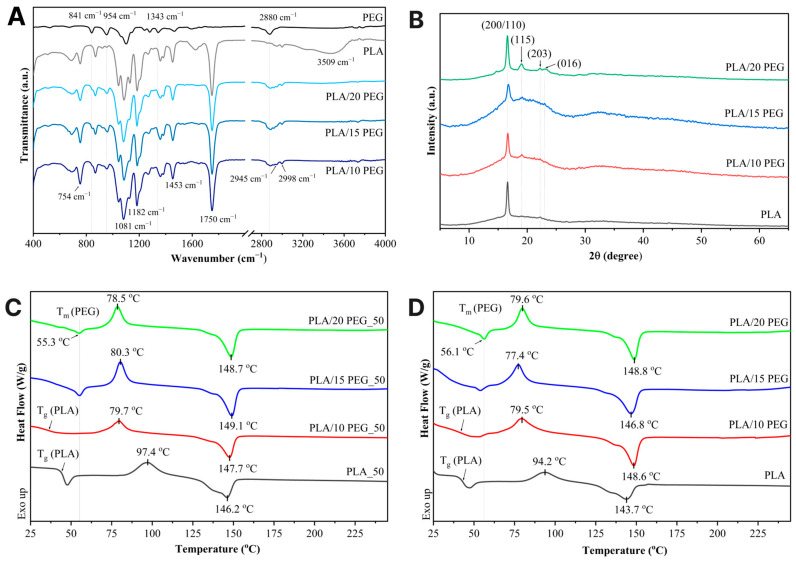
(**A**) ATR–FTIR spectra, (**B**) XRD patterns, (**C**,**D**) DSC curves of PLA and PLA/PEG scaffolds.

Top–view SEM images (Figure 1K,O) reveal that pure PLA has a textured surface with globular domains approximately 2–3 μm in size. Incorporation of PEG into the PLA matrix promoted the formation of a smoother scaffold surface (Figure 1L–N). EDS mapping (Figure S2) confirmed the uniform distribution of the C and O elements across the scaffold surface.The molecular structure and intermolecular interactions in the PLA/PEG scaffolds were investigated by ATR–FTIR analysis, as shown in Figure 2A. Pure PLA exhibited characteristic bands consistent with previous reports [30], including a strong C=O stretching vibration at 1750 cm^−1^ and a C–O–C stretching vibration at 1081 cm^−1^ associated with the PLA ester group. Additionally, the peaks at 2945 cm^−1^ and 2998 cm^−1^ correspond to the symmetric and asymmetric stretching vibrations of the CH_3_ group, respectively.

Upon PEG incorporation, the PLA’s C=O band at 1750 cm^−1^ remained unchanged, confirming the preservation of the PLA backbone. At the same time, distinct PEG–related bands appeared at 841 cm^−1^ (CH_2_ rocking vibrations), 954 cm^−1^ (CH_2_ rocking modes), 1343 cm^−1^ (CH_2_ wagging vibrations), and 2880 cm^−1^ (asymmetric CH_2_ stretching), with their intensities progressively increasing with PEG content [31]. In particular, the band at 954 cm^−1^ intensified, whereas the PLA band at 1182 cm^−1^, corresponding to the C–O–C stretching of the ether group and C–O stretching of the ester group, weakened. This suggests a greater effect of plasticization, with more PEG units between the PLA–PLA chains [9].

The XRD patterns (Figure 2B) show that all scaffolds exhibited dominant diffraction peaks at 2θ = 16.7° and 22.3°, which correspond to the (200)/(110) and (203) planes of the α crystal form of PLA. In PLA/PEG scaffolds, additional PEG diffraction peaks appeared at 19° and 23°, corresponding to the (115) and (016) planes of PEG [32]. The crystallite size was 56 nm for PLA, 55 nm for both PLA/10 PEG and PLA/15 PEG, and decreased to 38 nm for PLA/20 PEG. This may be related to PEG plasticization, enhancing chain mobility while restricting crystallite growth.

DSC was employed to investigate the thermal behavior of PLA and PLA/PEG scaffolds. Representative thermograms are shown in Figure 2C,D, and the corresponding thermal parameters and crystallinity are summarized in Table 2.

All scaffolds exhibited the typical thermal transitions of semicrystalline PLA. As shown in Figure 2C,D, the thermograms for PLA and PLA/PEG scaffolds demonstrate cold crystallization of the PLA phase above the *T*_g_, followed by the melting of PLA crystals at higher temperatures [33].

Incorporation of 10 wt.% PEG reduced *T*_g_ of PLA to 36.2–39.4 °C (Figure 2C,D, Table 2), which is close to the physiological temperature and therefore favorable for shape-memory recovery in minimally invasive bone scaffolds without inducing thermal damage to surrounding tissues. At higher PEG contents (15 wt.% and 20 wt.%), *T*_g_ could not be clearly identified in the measured temperature range due to overlapping PEG melting transitions [21]. At 10 wt.% PEG, the polymer is likely to be molecularly dispersed within the amorphous PLA domains, thereby lowering the *T*_g_ without producing a distinct PEG melting peak. At higher PEG contents, the solubility of PEG in PLA is exceeded, resulting in crystallite formation and phase separation, as confirmed by the appearance of PEG melting transition in DSC curves (Figure 2C,D) and PEG diffraction peaks in XRD patterns (Figure 2B).

The addition of PEG also affected the crystallization behavior of PLA. The *T*_cc_ decreased from 94.2 °C for pure PLA to 77.4–79.6 °C in PLA/PEG scaffolds. *T*_m_ was increased in PLA/PEG scaffolds, suggesting the formation of more stable or differently ordered PLA crystallites affected by PEG [34]. *X*_c_ increased with PEG content, rising from 7.4% in pure PLA to 11.2% in PLA/20 PEG for scaffolds with 100% infill density. A similar trend was noted for PLA/PEG_50 gyroid scaffolds (Table 2).

The reduction in *T*_g_ and *T*_cc_, along with the rise in *X*_c_ as the PEG content increased, is attributed to the enhanced flexibility of PLA chains resulting from the plasticizing effect of PEG [35]. By diffusing into the PLA matrix, PEG molecules insert themselves between the polymer chains, expanding the free volume and weakening interchain interactions. This facilitates greater chain mobility, allowing the PLA to move more freely at lower temperatures.

Notably, the *X*_c_ values represent the crystalline fraction present after FFF processing and can therefore reflect the printing thermal history, including cooling and layer-by-layer reheating. Nevertheless, the PEG-dependent decrease in *T*_cc_ and the increase in *X*_c_ observed for PLA/PEG and PLA/PEG_50 scaffolds (despite printing at a lower nozzle temperature than neat PLA) are consistent with previous studies on PLA/PEG blends, which demonstrate that PEG enhances PLA chain mobility and accelerates crystallization.

**Figure 3:**
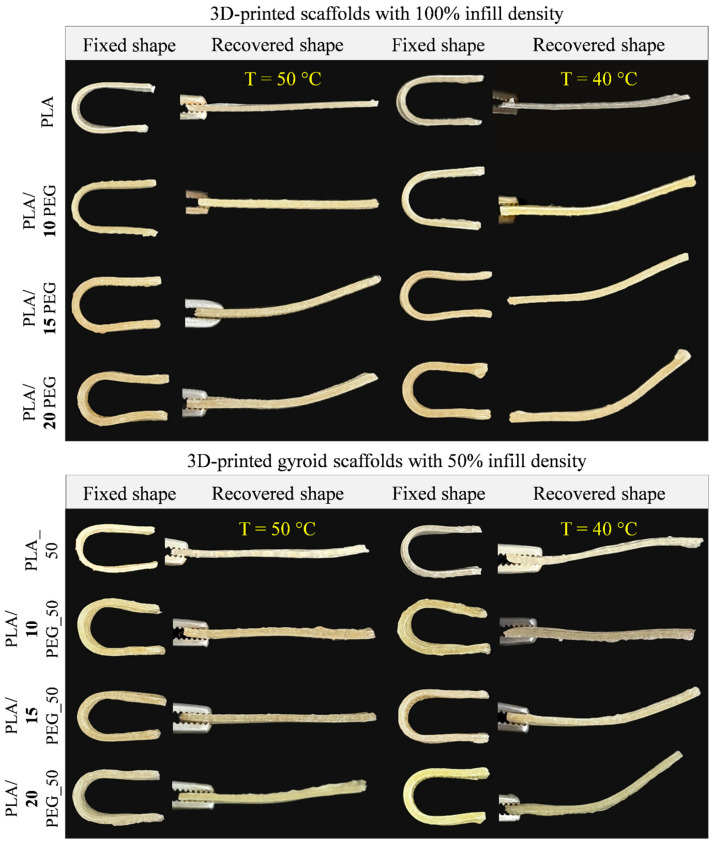
Photographs of 3D-printed scaffolds at fixed and recovered shapes at different activation temperatures.

The shape-memory performance of 3D-printed scaffolds was evaluated at two activation temperatures (40 °C and 50 °C), and the corresponding *R*_f_, *R*_r_, and *T* values are summarized in Table 3. Representative photographs and videos of the shape-memory process are shown in Figure 3 and Videos S1–S4.

In thermally activated SMPs, the activation temperature is the actual temperature at which the trigger shape recovery occurs. As noted, this is typically above *T*_g_ (e.g., for amorphous SMP) or at the melting point of crystals (e.g., for PCL-based SMP). In either case, reaching this temperature allows the chains to move and the stored elastic energy to drive recovery. The closer the activation temperature is to the polymer’s *T*_g_, the lower the energy input required. However, if the activation temperature is set very high (far above *T*_g_), the SMP will recover more vigorously but may also suffer from thermal expansion or degradation that can actually reduce the net recovery ratio. It is therefore important to choose an activation temperature that is high enough to fix the temporary shape but low enough to allow biomedical actuation.

In this study, all scaffolds exhibited complete fixation of the temporary shape (*R*_f_ = 100 ± 1%). At an activation temperature of 50 °C, scaffolds exhibited rapid and efficient shape recovery (*R*_r_ > 87% and *T* < 12 s). For the scaffolds with 100% infill, the *R*_r_ decreased progressively with increasing PEG content (Table 3), particularly at 20 wt.% PEG, where smaller crystallites (38 nm) and phase-separated PEG-rich domains compromise the continuity of the switching network, dissipating stored elastic energy and reducing recovery efficiency [16]. Additionally, the high *R*_r_ for PLA scaffolds can be attributed to the relatively high fraction of amorphous regions in pure PLA, which allows chains to return more readily to a random coil state upon heating [36].

In contrast, all gyroid scaffolds retained high *R*_r_. This enhanced response is attributed to the gyroid structure, which provides a large specific surface area and more uniform penetration of heat and water during the thermal activation, thereby facilitating a faster and more complete recovery of the entire scaffold. At higher temperatures, the increased segmental mobility above the *T*_g_ compensates for the PEG-induced morphological heterogeneity, explaining the improved recovery observed for PLA/15 PEG_50 and PLA/20 PEG_50 upon heating at 50 °C.

At 40 °C, shape recovery was slower due to reduced thermal activation, with *T* extending to 6–8 min (Table 3). *R*_r_ decreased progressively with increasing PEG content. An exception to this general trend is observed for the PLA/10 PEG_50 gyroid scaffold, which exhibits a high recovery ratio (*R*_r_ = 97 ± 1%) at 40 °C with a *T* = 6 ± 1 min. This enhanced performance can be attributed to a combination of optimized thermal properties and structural effect at 10 wt.% PEG, uniform plasticization of the PLA matrix is achieved without pronounced phase separation, resulting in a reduced glass transition temperature (*T*_g_ = 36.2 °C), which enables effective shape-memory activation near physiological temperature. Thus, the PLA/10 PEG_50 was identified as the optimal material for shape-memory tissue engineering applications.

**Figure 4:**
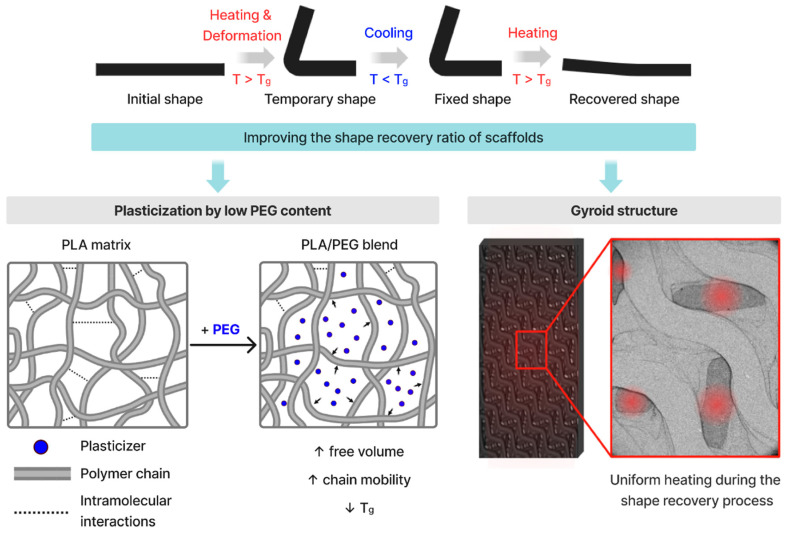
Illustration of the influence of plasticizer and gyroid structure on the SME (The arrow indicates the plasticisation of the PLA matrix induced by PEG addition, which increases the free volume between PLA polymer chains.).

Additionally, the gyroid architecture plays a critical role in facilitating recovery at low activation temperatures. The gyroid structure provides a high specific surface area and interconnected pathways, promoting more uniform heat transfer throughout the scaffold during thermal activation (Figure 4). Although the effective thermal conductivity of the printed scaffolds was not directly measured, previous quantitative studies on TPMS lattices demonstrate that gyroid topologies exhibit superior heat-transfer efficiency compared to conventional architectures. This combination of reduced *T*_g_ and efficient heat distribution enables rapid and homogeneous segmental mobilization, resulting in near-complete shape recovery of PLA/10 PEG_50 at 40 °C.

For instance, PLA and PLA_50 exhibited high *R*_r_ values at 50 °C and 40 °C. These results indicate that for scaffolds without PEG, the shape-memory performance is robust and largely independent of scaffold architecture or moderate changes in activation temperature, highlighting the intrinsic ability of PLA chains to return to their initial configuration efficiently.

The effect of PEG on shape-memory behavior can be understood by considering its role in the PLA matrix. PEG acts as a plasticizer, increasing chain mobility in the amorphous regions and lowering the *T*_g_ of PLA (Figure 4). These plasticized amorphous phase function as the reversible switching phase, freezing below *T*_g_ to fix the temporary shape and mobilizing above *T*_g_ to enable entropic elastic recovery. PLA crystallites constitute the permanent phase (netpoints), providing structural stability and the restoring force required for macroscopic recovery.

While PEG addition increases overall crystallinity and accelerates crystallization due to enhanced chain mobility, it also leads to a reduction in crystallite size, particularly at 20 wt.% PEG. Excessive PEG therefore promotes phase separation and the formation of PEG-rich domains and refined crystallites, which act as structural defects and weaken the integrity of the switching phase.

In practice, increasing crystallinity tends to raise recovery stress but can lower the shape recovery ratio if chains cannot fully disengage. Nie et al. found that annealing a semicrystalline PLA produced more perfect crystals and higher overall crystallinity, and that these crystalline regions became the netpoints governing shape-memory performance [37]. As a result, longer annealing (more crystallinity) reduced the recovery ratio while increasing the recovery stress.

Exposure to physiological conditions makes the PLA/PEG scaffolds sensitive to PEG-dependent water transport and morphology, which are crucial to long-term stability and biodegradation. PEG increases hydrophilicity and water accessibility in PLA-based materials. Studies on 3D-printed PLA/PEG scaffolds report that PEG-containing materials show faster in vitro degradation than neat PLA, accompanied by PEG-dependent changes in surface and geometry that are relevant for property retention over time [7]. PLA degradation is further governed by crystallinity and construct geometry, which regulate diffusion paths and the accessibility of ester bonds to hydrolysis under physiological conditions [38]. PEG molecular weight is also a meaningful variable in PLA/PEG architectures. PLA–PEG–PLA degraded in phosphate-buffered saline at 37 °C shows composition-dependent swelling and mass-loss kinetics, with the PEG segment molecular weight influencing water uptake and degradation rate [39]. Degradation studies of PEG-plasticized PLA also show peak-type water absorption followed by mass loss in PEG-containing materials, consistent with PEG-related mass-loss and migration processes that can alter the composition during exposure [40].

A related biological concern for PLA implants is local acidification from degradation products. In an in vitro model near degrading PLA-PGA implants, the surrounding medium pH dropped substantially over time (down to 3.0 at 9 weeks), illustrating the potential for an acidic microenvironment at the implant interface [41]. Interfacial pH shifts have been shown to influence osteoblast behavior on acidic degradable polymer surfaces, supporting the relevance of local pH control for bone tissue engineering applications [42]. A recent study also links PLA degradation products to immune-cell activation pathways associated with chronic inflammation and fibrosis, underscoring why PEG-induced acceleration of PLA hydrolysis should be considered when developing PLA/PEG implants [43].

PEG concentration and molecular weight are also routinely used as design variables in PLA implants as they influence hydration, degradation kinetics, and interface biology. In PLLA/PEG scaffolds, PEG content was evaluated in relation to degradation rate and biocompatibility endpoints (including cytotoxicity and irritation-related tests), demonstrating PEG-dependent biological performance [44]. In biodegradable PLLA/PDLLA blends plasticized with PEG 400 (4–12 wt.%), a higher PEG content resulted in higher in vitro weight loss during immersion, indicating PEG-level control over degradation behavior under aqueous exposure [45].

Additionally, the cytotoxicity of PLA and PLA/PEG scaffolds was studied in vitro using direct contact and incubating cells with extracts from the materials in a nutrient medium, as described in the Supplementary Information. Compared to the control group, cell viability in the samples was 40% lower after 24 h of incubation (Figure S5A). Cell viability after 24 h of culturing can be correlated with the initial number of cells attached to the substrate. However, the surface of the plate plastic is specially treated to enhance cell adhesion, making it easier for cells to attach to the substrate, divide, and migrate. These results indicate that PEG in the PLA composition has no adverse effect on fibroblast proliferation. Extracts from the materials after 48 h of exposure showed no cytotoxic effect against 3T3 (Figure S5B), further demonstrating that PEG does not affect the biocompatibility of PLA scaffolds.

## 4. Conclusions

The plasticizer content and scaffold structure influence the shape-memory behavior of PLA/PEG scaffolds. All scaffolds exhibited complete *R*_f_, whereas *R*_r_ and *T* depended on activation temperature, PEG content, and infill density.

Incorporation of 10 wt.% PEG reduced the *T*_g_ of PLA and promoted crystallization, facilitating low–temperature activation of the SME. Higher PEG content (15 wt.% and 20 wt.%) induced phase separation, which adversely affected shape recovery efficiency, particularly at 40 °C. Shape recovery of scaffolds in water medium at 40 °C was limited due to restricted PLA chain mobility, whereas at 50 °C, increased PLA chain mobility within the same structure resulted in substantially faster and more complete shape recovery.

Notably, the PLA/10 PEG_50 gyroid scaffold demonstrated the best shape-memory performance, achieving both *R*_r_ = 97 ± 1% at 50 °C and 40 °C. This is attributed to efficient plasticization with improved heat transfer provided by its interconnected gyroid porous network. These findings highlight the critical interplay between material composition, plasticization, and 3D-printed structural design in tuning the shape-memory properties of PLA/PEG scaffolds for potential biomedical applications.

Future work should quantify compressive and fatigue performance under bone-relevant conditions to determine how PEG content and gyroid structure affect load bearing. Exploring alternative PEG molecular weights, compositions, and TPMS designs, together with in vitro and in vivo tests, will help define design parameters that balance shape-memory behavior, mechanical integrity, and biological response.

## Figures and Tables

**Table 1:** Sample codes, compositions, and infill densities of PLA and PLA/PEG scaffolds.

Sample Code	Mass Fraction of PLA (wt.%)	Mass Fraction of PEG (wt.%)	Infill Density (%)
PLA	100	0	100
PLA/10 PEG	90	10	100
PLA/15 PEG	85	15	100
PLA/20 PEG	80	20	100
PLA_50	100	0	50 (gyroid)
PLA/10 PEG_50	90	10	50 (gyroid)
PLA/15 PEG_50	85	15	50 (gyroid)
PLA/20 PEG_50	80	20	50 (gyroid)

**Table 2:** Thermal properties and crystallinity of 3D-printed scaffolds.

Sample	*T*_g_ (°C)	*T*_cc_ (°C)	*T*_m_ (°C)	Δ*H*_cc_ (J/g)	Δ*H*_m_ (J/g)	*X*_c_ (%)
PLA	42.2	94.2	143.7	17.0	23.9	7.4
PLA/10 PEG	39.4	79.5	148.6	16.0	22.7	8.0
PLA/15 PEG	–	77.4	146.8	17.3	24.3	8.9
PLA/20 PEG	–	79.6	148.8	14.6	22.9	11.2
PLA_50	43.9	97.4	146.2	17.4	22.5	5.5
PLA/10 PEG_50	36.2	79.7	147.7	17.1	23.9	8.1
PLA/15 PEG_50	–	80.3	149.1	16.9	25.2	10.5
PLA/20 PEG_50	–	78.5	148.7	14.9	24.6	13.0

**Table 3:** Shape-memory behavior of the PLA and PLA/PEG scaffolds.

Sample	Activation Temperature: 50 °C			Activation Temperature: 40 °C		
	*R*_f_ (%)	*R*_r_ (%)	*T* (s)	*R*_f_ (%)	*R*_r_ (%)	*T* (min)
Scaffolds with 100% infill density						
PLA	100 ± 1	97 ± 2	8 ± 1	100 ± 1	93 ± 2	6 ± 1
PLA/10 PEG	100 ± 1	92 ± 4	6 ± 1	100 ± 1	88 ± 2	6 ± 1
PLA/15 PEG	100 ± 1	87 ± 5	7 ± 1	100 ± 1	87 ± 7	6 ± 1
PLA/20 PEG	100 ± 1	89 ± 2	8 ± 1	100 ± 1	79 ± 3	7 ± 1
Gyroid scaffolds with 50% infill density						
PLA_50	100 ± 1	97 ± 3	6 ± 1	100 ± 1	94 ± 5	6 ± 1
PLA/10 PEG_50	100 ± 1	97 ± 1	6 ± 1	100 ± 1	97 ± 1	6 ± 1
PLA/15 PEG_50	100 ± 1	98 ± 1	10 ± 1	100 ± 1	87 ± 3	8 ± 1
PLA/20 PEG_50	100 ± 1	98 ± 2	12 ± 1	100 ± 1	80 ± 3	8 ± 1

## Data Availability

The original contributions presented in this study are included in the article/Supplementary Materials. Further inquiries can be directed to the corresponding author.

## Supplementary Materials

The following supporting information can be downloaded at: https://www.mdpi.com/article/10.3390/polym18010140/s1, Figure S1: Slicer preview of the gyroid TPMS architecture used for printing (50% infill); Figure S2: SEM micrographs, elemental mapping, and EDS spectra of PLA (**A**–**D**), PLA/10 PEG (**E**–**H**), PLA/15 PEG (**I**–**L**), and PLA/20 PEG (**M**–**P**) scaffolds; Figure S3: Raman spectra of PEG flakes, PLA and PLA/PEG scaffolds; Figure S4: DSC curves of PLA and PLA/PEG scaffolds; Figure S5: (**A**) Viability of 3T3 cells after 24 h of direct contact with the test samples and (**B**) cytotoxicity of extracts from the material after 48 h of incubation in the nutrient medium. * Statistically significant differences at *p* < 0.01 compared to the control group after 24 h of incubation. Table S1: FFF 3D printing parameters for PLA and PLA/PEG scaffolds; Table S2: Comparison of materials with low-temperature shape-memory effect. References [46,47,48,49,50] are cited in the supplementary materials. Videos S1–S4 can be downloaded at: https://wdfiles.ru/2Qw3u, https://wdfiles.ru/2Qw3x, https://wdfiles.ru/2Qw3y, https://wdfiles.ru/2Qw3z. Video S1. Shape-memory recovery of PLA and PLA/PEG scaffolds (100% infill) at 50 °C. Video S2. Shape-memory recovery of PLA and PLA/PEG scaffolds (100% infill) at 40 °C. Video S3. Shape-memory recovery of PLA and PLA/PEG scaffolds (50% gyroid infill) at 40 °C. Video S4. Shape-memory recovery of PLA and PLA/PEG scaffolds (50% gyroid infill) at 50 °C.
