# Supplementary material for: 3D-Printed Poly(lactic acid)/Poly(ethylene glycol) Scaffolds with Shape-Memory Effect near Physiological Temperature
Source: Polymers (Basel). 2026 Jan 3;18(1):140. doi: 10.3390/polym18010140 (PMC12787712; doi:10.3390/polym18010140)
Supplement: Supplementary file 1 [file polymers-18-00140-s001.zip › polymers-4053270-supplementary.pdf]

Supplementary Information

3D-Printed Poly(lactic acid)/Poly(ethylene glycol) Scaffolds with Shape-Memory Effect near Physiological Temperature

Anastasia A. Fetisova [1,2], Abdullah bin Firoz [2], Alexandr S. Lozhkomoev [2,3], Elena I. Senkina [3], Egor E. Ryumin [3], Maria A. Surmeneva [1,2] and Roman A. Surmenev [1,2,*]

[1] International Research and Development Center Piezo- and Magnetoelectric Materials, Research School of Chemistry and Applied Biomedical Sciences, National Research Tomsk Polytechnic University, 30 Lenina Avenue, Tomsk 634050, Russia
[2] Physical Materials Science and Composite Materials Centre, Research School of Chemistry and Applied Biomedical Sciences, National Research Tomsk Polytechnic University, 30 Lenina Avenue, Tomsk 634050, Russia
[3] Institute of Strength Physics and Materials Science of Siberian Branch of the Russian Academy of Sciences (ISPMS), 2/4 Akademicheskii Pr., Tomsk 634055, Russia
* Correspondence: rsurmenev@mail.ru

S1. Materials and Methods

Additively manufactured samples featuring a gyroid triply periodic minimal surface (TPMS) structure were produced using fused filament fabrication (FFF) on an Ultimaker 3 3D printer. The printing parameters are summarized in Table S1. A representative slicer preview of the 50% gyroid infill is provided in Figure S1, and the corresponding gyroid design file (STL/3D model).

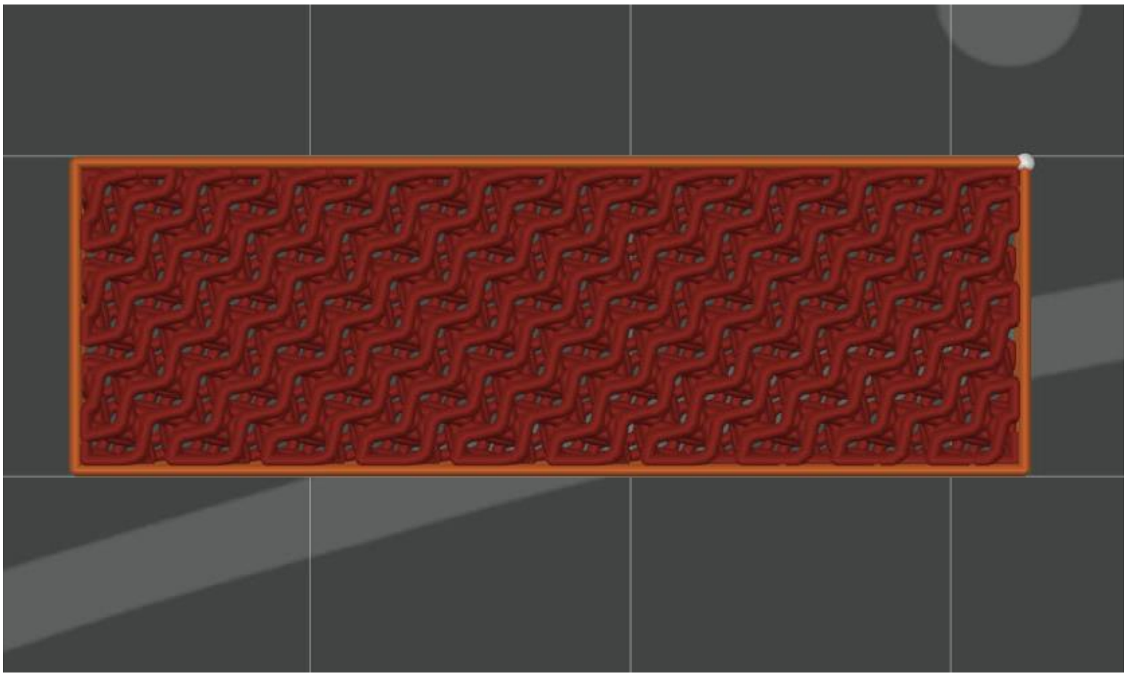

Figure S1. Slicer preview of the gyroid TPMS architecture used for printing (50% infill).

Table S1. FFF 3D printing parameters for PLA and PLA/PEG scaffolds.

Parameters/Sample	PLA	PLA/PEG
Nozzle temperature (°C)	190	180
Nozzle diameter (mm)	0.8	0.8
Bed temperature (°C)	60	60
Layer thickness (mm)	0.2	0.2
Printing speed (mm/s)	40	40
Flow rate (%)	100	100

Raman spectra were recorded using an integrated system comprising a vertical optical microscope with a 20× objective and an integrated atomic force microscopy head (NTEGRA, NT–MDT, Russia), coupled to a Raman spectrometer (nVia Raman Micro-scope, Renishaw, UK) with a 532 nm He–Ne laser, and an Andor detector (1024 × 400 pixels CCD).

Thermal properties of scaffolds were studied by differential scanning calorimetry (DSC, Q2000 V24.10 Build 122, TA Instruments, USA). Samples of approximately 8 mg taken from the cross–section of scaffolds were sealed in aluminum pans and scanned from 25 °C to 250 °C at a heating rate of 5 °C/min under continuous air flow (60 mL/min).

Cytotoxicity of the samples was studied by direct contact and by incubating cells with extracts from the materials in a nutrient medium. Mouse fibroblasts of the 3T3 line were used for the experiment. Cells were cultivated in a DMEM/F-12 nutrient medium (BioloT, Russia) supplemented with fetal bovine serum (FBS) at 10 wt.% (BioloT, Russia) and an antibiotic (streptomycin) at 5 wt.% (BioloT, Russia). A Sanyo MCO-5AC incubator (Sanyo, Japan) with a constant temperature of 37 °C and an air atmosphere containing 5% CO_2 was used. Cell viability was studied using the standard MTT test protocol (ISO 10993-5, ISO 10993-12). The optical density of dissolved formazan was determined using a Multiscan FC photometer (Thermo Scientific, Germany) at a wavelength of 620 nm.

Sterile material samples measuring 10 × 10 × 2 mm^3 were placed into the wells of a 24-well plate. Sterilization was accomplished by soaking the samples in 70% alcohol for 1-2 minutes. After the samples were completely dry, 2 mL of complete nutrient medium was added to each well to remove residual solvent. The plate with the samples was placed in an incubator for 48 h. After two days of incubation, the nutrient medium was removed from the wells. Next, a drop of cell suspension at a concentration of 0.5×10^5 cells/mL was applied to the surface of the samples. The plates were placed in the incubator for 5-7 minutes to allow

primary cell adhesion, preventing them from being immediately washed off the samples after adding the nutrient medium. After 5-7 minutes, 1 mL of nutrient medium was added to the wells. A plastic plate was used as a control, and an MTT assay and viable cell count were performed on it.

Sterile material samples measuring 8 × 80 × 1 mm^3 were placed into the wells of a 24-well plate, and 2.5 mL of DMEM/F12 nutrient medium was added to each well. The plates with samples were then kept at 37 °C for 48 h. Twenty-four hours before the end of extraction, 3T3 cell culture was passaged into the wells of a 96-well plate at 10×10^3 cells. The volume of liquid in the wells of the 96-well plate was 100 µL. After 24 h, the nutrient medium in the wells with cells was replaced with extracts from the studied samples at concentrations of 25%, 50%, 75%, and 100%. Extracts were also diluted in DMEM/F12 nutrient broth. After changing the medium in the wells, the plates were incubated for 24 hours at a constant temperature of 37 °C and an air atmosphere of 5% CO_2. Following incubation, an MTT assay and viable cell count were performed.

To perform the MTT assay, 100 µL of 3-4,5-dimethylthiazole-2,5-diphenyl tetrazolium bromide (MTT solution 5 mg/mL) were added to the medium. The mixture was incubated for 4 hours at 37 °C with 5% CO_2. The MTT reagent, under the action of mitochondrial dehydrogenases in viable cells, was converted into water-insoluble formazan, which has a violet color. After removing the resulting mixture, 1 mL of dimethyl sulfoxide (DMSO) was added to the wells. The formazan crystals dissolve within 15 minutes. To determine the optical density of the dissolved formazan, triplicate 100 µL aliquots of the solution were taken from the original wells and transferred to a 96-well plate. Cell number was determined using calibration graphs of the dependence of optical density on cell concentration. The significance of differences in cell optical density in experimental wells compared to control wells was determined using the Mann-Whitney U test. The data presented as mean ± standard deviation. Differences were considered statistically significant at $p < 0.01$.

S2. Results and Discussion

EDS analysis of PLA and PLA/PEG scaffolds revealed carbon and oxygen as the primary elements, fully consistent with the expected material composition (Figure S2). The copper peaks observed in spectra originate from the sputter-coating process and are not related to the studied materials. EDS mapping showed a uniform distribution of the detected elements across all scaffold surfaces.

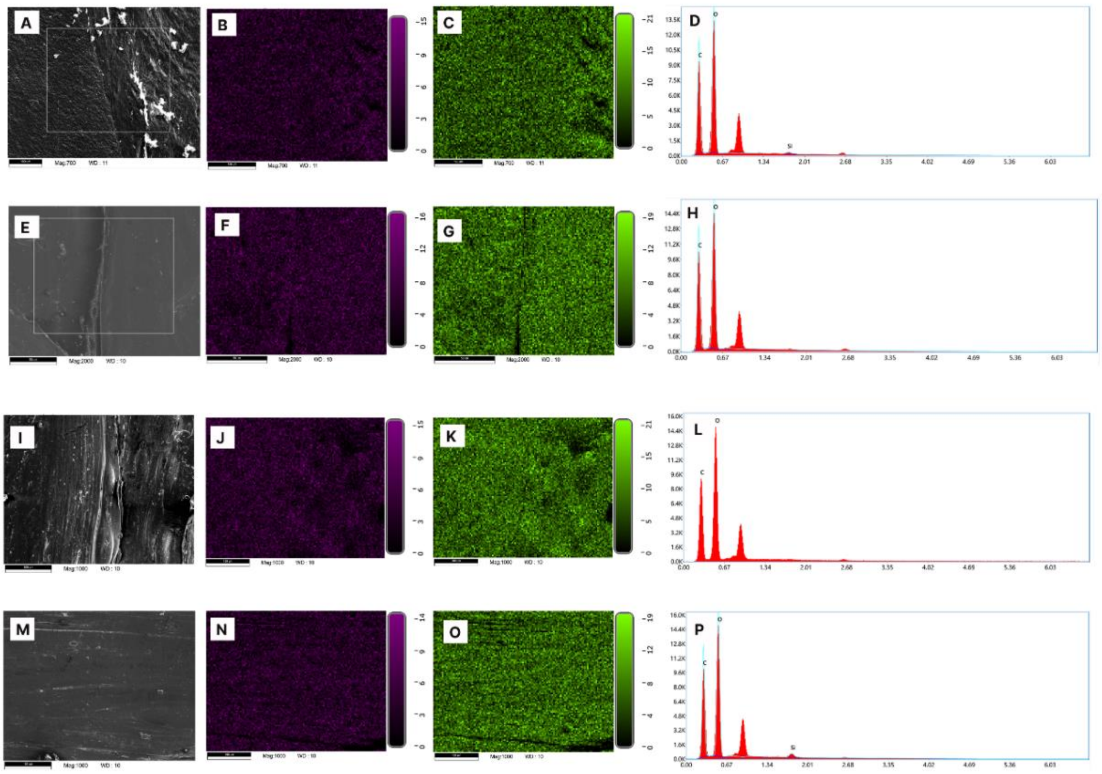

Figure S2. SEM micrographs, elemental mapping, and EDS spectra of PLA (**A**–**D**), PLA/10 PEG (**E**–**H**), PLA/15 PEG (**I**–**L**), and PLA/20 PEG (**M**–**P**) scaffolds.

The Raman spectra of PLA and PLA/PEG scaffolds (Figure S3) showed the characteristic bands of semicrystalline PLA at 874 cm^{-1}, 1044 cm^{-1}, 1127 cm^{-1}, 1299 cm^{-1}, 1454 cm^{-1} and 1771 cm^{-1}, assigned to C–COO stretching, coupled $\nu(C_{\alpha}–C_{\beta})/CH_3$ rocking, asymmetric CH_3 rocking, CH_2/CH_3 twisting, CH_3 bending, and C=O stretching, respectively [1, 2]. In PLA/15 PEG and PLA/20 PEG, additional PEG shoulders appeared at 1280 cm^{-1} and 1481 cm^{-1}, confirming PEG incorporation. The absence of peak shifts suggests that PEG is incorporated into PLA without any chemical interaction.

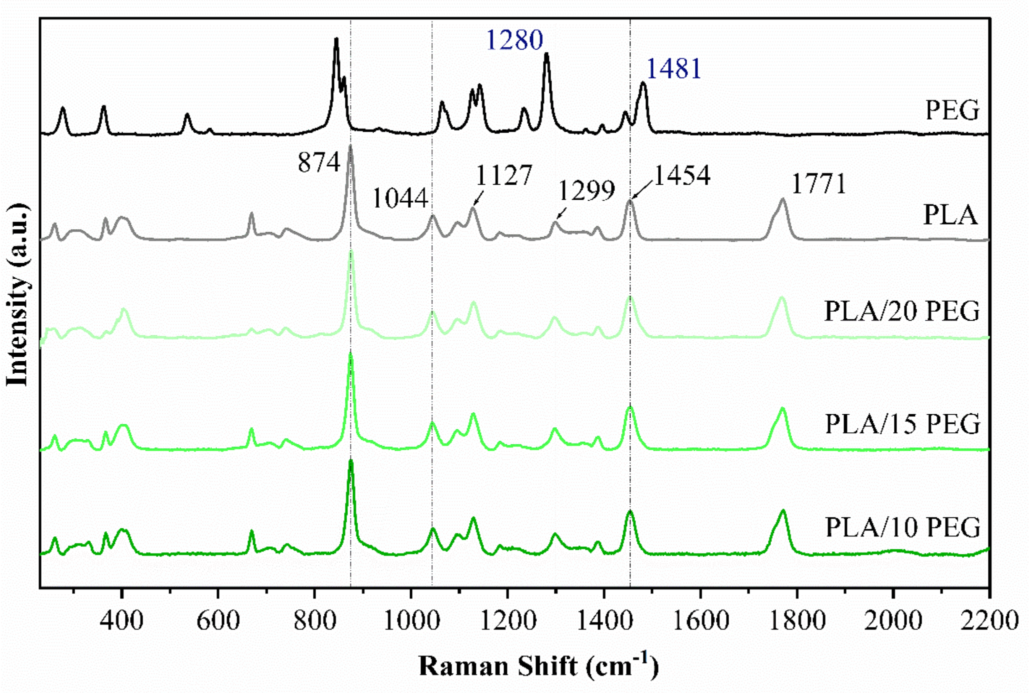

Figure S3. Raman spectra of PEG flakes, PLA and PLA/PEG scaffolds.

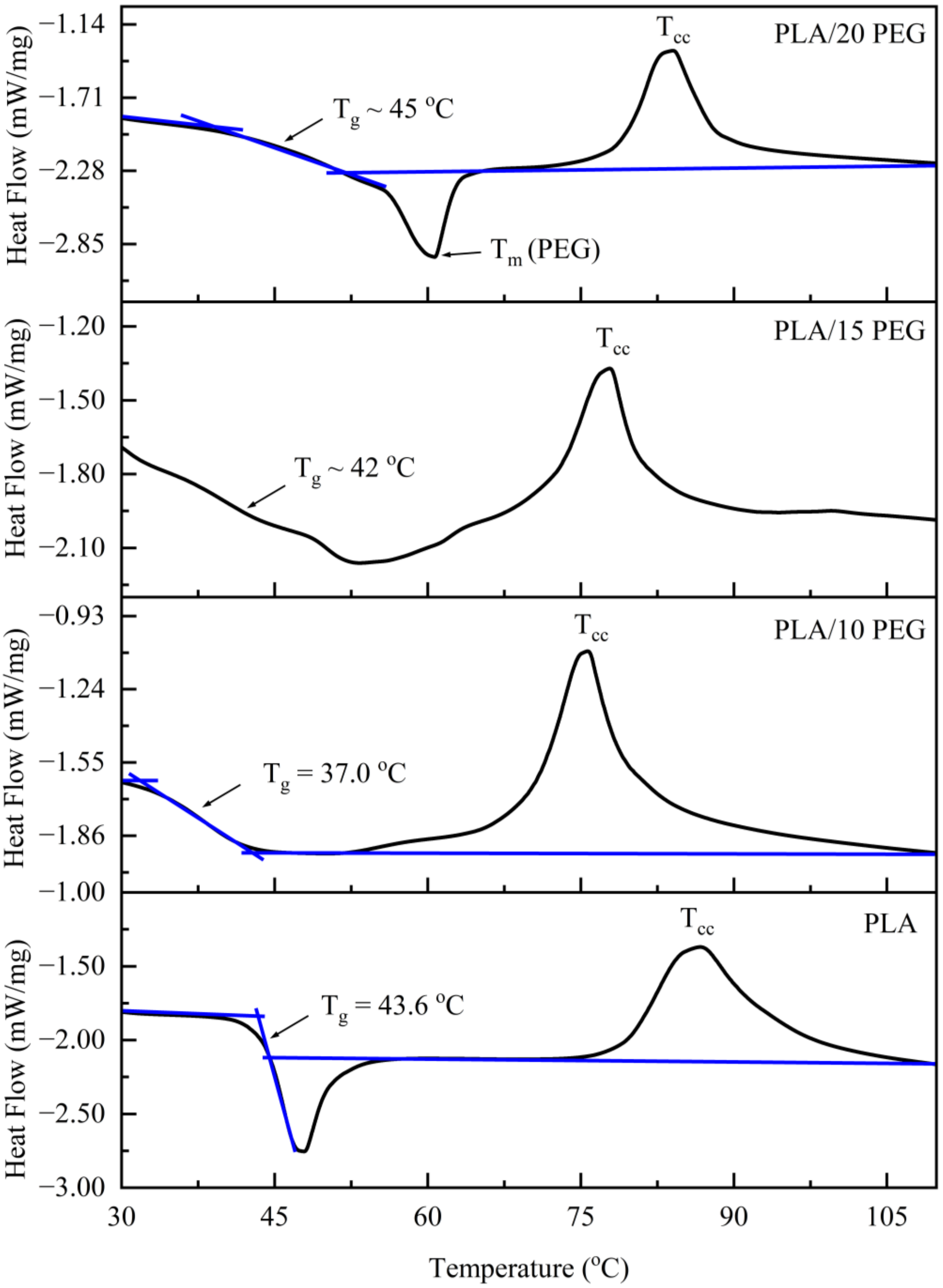

Figure S4. DSC curves of PLA and PLA/PEG scaffolds.

The DSC thermograms of PLA and PLA/PEG scaffolds (Figure S4), recorded at a reduced heating rate of 5 °C/min. The T_g of neat PLA was observed at 43.6 °C, while the incorporation of PEG modified the glass transition behavior, yielding T_g values of 37.0 °C for PLA/10 PEG, ~ 42 °C for PLA/15 PEG, and ~ 45 °C for PLA/20 PEG. For the blends, the glass transition became noticeably broader and its onset shifted to lower temperatures, beginning at approximately 30 °C, reflecting increased heterogeneity in segmental dynamics due to PEG incorporation.

All samples exhibited cold crystallization upon heating. Notably, in the PLA/20 PEG scaffold, a distinct PEG melting peak was observed, confirming phase separation at high PEG content. In this composition, the cold crystallization temperature shifted back toward values characteristic of neat PLA, suggesting that PEG-rich domains reduce the effective plasticization of the PLA matrix and partially restore its original crystallization behavior.

Table S2 provides a comparison of the shape-memory performance of the PLA/PEG scaffold with previously results reported in the literature [3-5]. In contrast to most of these systems, the approach proposed in this study offers a simple method for obtaining scaffolds exhibiting a low-temperature shape-memory effect. Notably, PLA/10 PEG_50 combines a gyroid porous architecture with a high recovery ratio at an activation temperature of 40 °C, without the need for copolymerization or chemical functionalization of PLA, thereby broadening its potential for biomedical applications.

Table S2. Comparison of materials with low-temperature shape-memory effect.

**Plasticizer**	**Polymer matrix**	**Fabrication method**	**Structure**	T_g **(°C)**	R_r **(%)**	**Medium temperature (°C)**	**Ref.**
PEG	PLA	FFF 3D printing	Orthogonal periodic porous	40.8	91.7	45	[3]
Tributyl citrate	PLA	FFF 3D printing	Ordered porous	40.2	92.8	45	[4]
Nanocellulose	Polyurethane	FFF 3D printing	Porous	33.6	98.9	55	[5]
PEG	PLA	FFF 3D printing	Gyroid	36.2	97.0	40	This work

The cytotoxicity of the PLA and PLA/PEG scaffolds was studied *in vitro* using direct contact and by incubating cells with extracts from the materials in a nutrient medium. It was found that PEG in the PLA formulation had no negative effect on cell proliferation. Extracts from the materials also had no cytotoxic effect, and the number of viable cells was similar to that of the control group.

Figure S5A shows the MTT assay results. Compared to the control group, after 24 h of incubation, cell viability in the test samples was approximately 40% lower. Cell viability after 24 h of culturing can be

correlated with the initial number of cells attached to the substrate. However, the surface of the plate plastic is specially treated to enhance cell adhesion, making it easier for cells to attach to the substrate, divide, and migrate. These results indicate that PEG in the PLA composition has no negative effect on fibroblast proliferation. Extracts from the materials after 48 h of exposure showed no cytotoxic effect against 3T3 (Figure S5B), further demonstrating that PEG does not affect the biocompatibility of PLA *in vitro*.

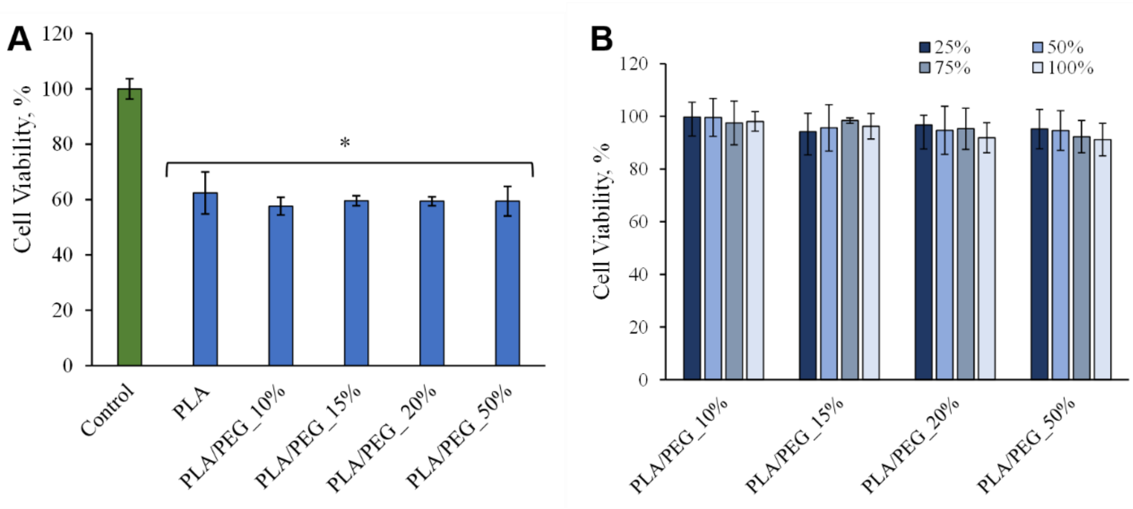

Figure S5. (**A**) Viability of 3T3 cells after 24 h of direct contact with the test samples and (**B**) cytotoxicity of extracts from the material after 48 h of incubation in the nutrient medium. * Statistically significant differences at $p < 0.01$ compared to the control group after 24 h of incubation.

References

1. Suzuki, T., et al., *Modification of physical properties of poly (L-lactic acid) by addition of methyl-β-cyclodextrin.* Beilstein journal of organic chemistry, 2014. **10**(1): p. 2997-3006.
2. Polak-Kraśna, K., et al., *Physical and mechanical degradation behaviour of semi-crystalline PLLA for bioresorbable stent applications.* Journal of the mechanical behavior of biomedical materials, 2021. **118**: p. 104409.
3. Guo, W., et al., *Dual Nano-Reinforced 3D-Printed Polylactic Acid Scaffolds for Antibacterial and Osteogenic Applications.* ACS Applied Nano Materials, 2025. **8**(20): p. 10471-10485.
4. Huang, Y., et al., *3D-Printed Thermally Activated Shape Memory PLA/TBC Composite Scaffold with Body-Compatible Temperature for Minimally Invasive Bone Repair.* ACS Applied Polymer Materials, 2025. **7**(7): p. 4572-4583.
5. Zhou, Y., et al., *3D printing of polyurethane/nanocellulose shape memory composites with tunable glass transition temperature.* Industrial Crops and Products, 2022. **182**: p. 114831.
